# Temple syndrome in Chinese children: a case series and literature review

**DOI:** 10.3389/fendo.2026.1871637

**Published:** 2026-10-19

**Authors:** Yeqing Yuan, Yunchong Chen, Ziyang Zhu, Chen Liu, Bixia Zheng, Wei Zhou, Chunli Wang, Wei Gu, Su Wu

**Affiliations:** 1 Department of Endocrinology, Children’s Hospital of Nanjing Medical University, Nanjing, China; 2 Department of Neonatology, Children’s Hospital Affiliated to Shandong University, Jinan, Shandong, China; 3 Nanjing Key Laboratory of Pediatrics, Children’s Hospital of Nanjing Medical University, Nanjing, China

**Keywords:** blended phenotypes, precocious puberty, rhGH therapy, short stature, Temple syndrome

## Abstract

**Introduction:**

Temple syndrome (TS14) is a rare imprinting disorder caused by aberrations at chromosome 14q32, manifesting as pre- and postnatal growth retardation, delayed motor milestones, and central precocious puberty (CPP). No cohort studies have characterized TS14 in Chinese children.

**Methods:**

Six patients from two tertiary hospitals in China were included in this study. All patients underwent molecular testing using whole exome sequencing (WES) and methylation-specific multiplex ligation-dependent probe amplification (MS-MLPA). Their clinical and molecular characteristics were systematically described. Two patients received recombinant human growth hormone (rhGH) therapy, one of whom was treated with long-acting GH. In addition, a structured literature review of TS14 was performed.

**Results:**

Six patients, all born small for gestational age and exhibiting persistent postnatal growth retardation, were molecularly diagnosed with TS14. In Patient 3, negative WES findings combined with an abnormal MS-MLPA result supported a methylation abnormality as the underlying mechanism. Patient 1 presented with CPP, and patient 3 with rapidly progressive early puberty, whereas patients 4–6 harbored additional molecular findings, including a *de novo DYNC1H1* deletion, a *YY1*-region deletion, and a homozygous *GALC* c.1901T>C variant; the findings in patients 4 and 5 may have contributed to blended phenotypes. In patient 1, combined gonadotropin-releasing hormone analog (GnRHa) and rhGH therapy resulted in a height gain of +1.0 standard deviation scores (SDS) over 1.5 years, while patient 3, who received long-acting GH, achieved a height gain of +1.74 SDS over 2.5 years. A review of 167 TS14 cases showed broadly similar core clinical features across ethnicities, although Asian patients exhibited lower rates of facial dysmorphism and obesity. Additional molecular findings, including multilocus imprinting abnormalities and potential blended phenotypes, were also identified in reported cases. Adult heights as low as −3.3 SDS were reported in untreated patients, whereas rhGH ± GnRHa improved growth outcomes but did not consistently normalize adult height.

**Conclusion:**

This series broadens the clinical characterization of TS14 in Chinese children and highlights the diagnostic value of comprehensive molecular testing, particularly in patients with atypical features. Hormonal treatment may improve growth velocity in selected patients, but long-term benefits, final height outcomes and optimal timing remain uncertain.

## Introduction

Temple syndrome(TS14, OMIM#616222) is an exceptionally rare imprinting disorder caused by maternal uniparental disomy of chromosome 14 (UPD14mat), epimutations such as hypomethylation of the normally methylated paternal allele, or deletions affecting the paternally inherited imprinted region at 14q32.2 (1). The first case of TS14 was described by Temple et al. in 1991 (2), and to date, over 100 cases have been reported in the literature.

Clinical recognition of TS14 remains challenging because of its non-specific and variable phenotypic. Typical manifestations include intrauterine growth retardation (IUGR), small for gestational age (SGA), neonatal hypotonia, feeding difficulties, delayed motor milestones, small hands and feet, and a tendency toward overweight or obesity later in life. The syndrome is frequently underdiagnosed, largely due to its phenotype overlap with other imprinting disorders such as Prader-Willi syndrome (PWS) and Silver-Russell syndrome (SRS) (3, 4). Notably, central precocious puberty (CPP) has emerged as a distinguishing clinical hallmark of TS14, rarely observed in PWS or SRS.

With advances in molecular diagnostics, an increasing number of TS14 cases with complex or atypical phenotypes have been found to harbor additional molecular findings (5–7). The coexistence of multiple genetic defects may exaggerate, attenuate or modify the typical phenotype, further obscuring the clinical boundaries of TS14. Owing to this marked phenotypic heterogeneity and overlap with other imprinting disorders, many cases remain underrecognized or misdiagnosed. Although several international cohort studies have improved understanding of TS14, reports from Chinese populations are exceedingly limited, with only four published cases to date (8–11). This lack of awareness may delay molecular confirmation, especially in children presenting with SGA, short stature, or CPP.

Given the growth failure and frequent pubertal abnormalities observed in TS14, recombinant human growth hormone (rhGH) and gonadotropin-releasing hormone analogs (GnRHa) have been used in several reported cases, with short-term height gains documented in some patients (12–14). However, the existing evidence remains limited, heterogeneous, and largely derived from case reports, small case series, and regional cohorts, with no prospective controlled trials available to date. In a recent study by Ogawa et al., untreated TS14 patients achieved a near-final height of approximately −2.1 standard deviation scores (SDS), whereas rhGH-treated patients reached a slightly lower near-final height of −2.4 SDS (15). These findings raise questions regarding treatment response and highlight the need for further investigation. Currently, no consensus exists regarding the optimal hormonal management for TS14, and whether combination therapy confers sustained benefit on final adult height remains an unresolved clinical question. Furthermore, data on treatment outcomes among Chinese TS14 patients remain scarce, hindering the development of evidence-based clinical guidelines.

In this study, we describe six pediatric cases of TS14 from China and provide a structured review of the literature to enhance clinical recognition of its phenotypic spectrum, clarify the diagnostic pathway, and contribute preliminary observations on therapeutic interventions.

## Methods

### Patients and clinical evaluation

This retrospective study included six Chinese patients with a molecularly confirmed diagnosis of TS14. Patients 1–5 were recruited from the Children’s Hospital of Nanjing Medical University, and Patient 6 was recruited from the Children’s Hospital Affiliated to Shandong University, between April 2019 and September 2025. All consecutively diagnosed patients who met the inclusion criteria during the study period were included. No exclusion criteria were applied. Medical records were reviewed to obtain demographic information, perinatal history, anthropometric measurements, developmental features, treatment information, and follow-up data. This study was approved by the Institutional Ethics Committee of the Children’s Hospital of Nanjing Medical University (Ethics approval number: 202401001-1). The requirement for informed consent for participation and for the use of routinely collected clinical data was waived by the ethics committee. Written informed consent for publication of identifiable patient photographs was obtained from the patients’ parents. All procedures performed in this study were in accordance with the ethical standards of the institutional and/or national research committee(s) and with the Helsinki Declaration (as revised in 2013).

Height was measured and converted to SDS using the standardized growth curves for Chinese children and adolescents aged 0 to 18 years. Bone age was assessed from a non-dominant hand–wrist radiograph (conventionally the left hand) using the Greulich-Pyle (G-P) atlas method.

Short stature was defined as height below −2 SDS or below the 3rd percentile (−1.88 SDS) for same sex, age, and ethnicity in a comparable living environment, according to Chinese national growth references. SGA was defined as birth weight below the 10th percentile for same sex and gestational age, based on Chinese birth weight reference charts. One patient underwent GH stimulation testing at an external hospital using arginine and clonidine as stimulation agents; detailed procedural information was not available from the referring institution.

CPP was defined by all of the following criteria: (1) onset of secondary sexual characteristics before age 7.5 years in girls and 9 years in boys; (2) activated hypothalamic-pituitary-gonadal (HPG) axis confirmed by GnRH stimulation test, with a peak LH ≥5 IU/L (measured by immunochemiluminescence assay, ICMA) and a peak LH/FSH ratio ≥0.6; and (3) bone age advanced by ≥1 year relative to chronological age, with accelerated linear growth. Rapidly progressive early puberty (RPEP) was defined as pubertal onset after the CPP age cutoffs (girls ≥7.5 years, boys ≥9 years), accompanied by all of the following rapid progression markers: (1) biochemical HPG axis activation; (2) bone age advancement ≥2 years relative to chronological age with accelerated linear growth; and (3) rapid Tanner stage progression (advancement of one Tanner stage within 6 months).

rhGH was initiated at a dose of 0.15 IU/kg/day (0.05 mg/kg/day), within the recommended range for children born SGA. In patient treated with long-acting GH, a weekly dose of 0.2 mg/kg was administered, which is consistent with the current international consensus for long-acting GH therapy. GnRHa therapy was initiated in patients with CPP or RPEP in whom predicted adult height was compromised.

### Diagnostic algorithm

All patients in this study underwent a two-tier diagnostic workflow for the molecular evaluation of TS14. In the first (screening) tier, all enrolled patients underwent whole exome sequencing (WES) as the initial screening test, regardless of whether an imprinting disorder was initially suspected. In the second (confirmatory) tier, all patients with WES findings suggestive of UPD or 14q32 deletion, as well as those with negative WES results but persistent strong clinical suspicion of TS14, underwent methylation-specific multiplex ligation-dependent probe amplification (MS-MLPA). MS-MLPA was performed to quantitatively assess the methylation status of the 14q32 imprinting control region and to screen for copy number variations (CNVs).

### Genetic analysis

WES was performed for all probands, with trio-based WES (proband and parents) conducted in two cases. Genomic DNA was extracted from peripheral blood using the DNA isolation kit (Tiangen, China). Quality controlled-gDNA was hybridized with IDT’s xGen Exome Research Panel v1.0 probe capture array to enrich the DNA fragments of the target region and build a whole exon library. The enriched libraries were analyzed based on high-throughput sequencing by Illumina NovaSeq 6000 series sequencer (PE150). According to the sequence depth and variant quality, high quality and reliable variants were obtained. Pathogenicity analysis was performed according to the guidelines issued by the American College of Medical Genetics and Genomics (ACMG), with validation of the variants through Sanger sequencing.

### MS-MLPA

MS-MLPA was conducted using the ME032-B1 UPD7-UPD14 kit (MRC Holland, Amsterdam, The Netherlands). This assay contains 15 MLPA probes specific for sequences in the critical region of chromosome 14q32. DNA was processed in parallel with and without digestion with the methylation sensitive HhaI enzyme to detect both methylation deregulation and CNV. Data analysis was performed using the Coffalyser.net software v.240129.1959. For each experiment, reference samples were required to meet the manufacturer’s quality criteria (SD ≤ 0.10; FR 0.80–1.20). Copy number status was determined using the following FR cut-offs: normal (0.80 < FR < 1.20), heterozygous deletion (0.40 < FR < 0.65), and heterozygous duplication (1.30 < FR < 1.65). For the 14q32 imprinted probes (MEG3), methylation status was interpreted as: normal (0.40 ≤ FR ≤ 0.65), loss of methylation (FR < 0.40), or gain of methylation (FR > 0.65). Loss of methylation at MEG3:TSS-DMR, combined with either normal copy number (suggestive of maternal UPD14 or epimutation) or reduced copy number (suggestive of paternal 14q32 deletion), was required for the diagnosis of TS14. MS-MLPA alone cannot distinguish maternal UPD14 from primary epimutation. However, this distinction was resolved by analyzing WES-derived SNP data for regions of homozygosity (ROH) on chromosome 14. The presence of extended ROH spanning the 14q32 region indicated maternal UPD14, whereas the absence of ROH was consistent with a primary epimutation.

### Literature search

To characterize the clinical and molecular spectrum of TS14, we performed a structured literature search in PubMed, Web of Science, China National Knowledge Infrastructure (CNKI, cnki.net) and Wan fang Data (wanfangdata.com.cn) using the terms ‘Temple syndrome’, ‘TS14’ and ‘Temple syndrome 14’ from database inception to September 1, 2025. The search identified 338 records. After removal of 90 duplicates, 248 unique records underwent title and abstract screening, and 168 were excluded as irrelevant. Full texts of the remaining 80 articles were assessed, of which 47 were excluded because they lacked original patient-level data, provided insufficient clinical or molecular information, or were reviews without newly reported cases. Ultimately, 33 studies met the inclusion criteria. Eligible studies included case reports, case series, and cohort studies reporting individual-level clinical and/or molecular data from patients with molecularly confirmed TS14. Two authors independently screened records and assessed full texts, with disagreements resolved by consensus. To avoid duplicate counting, demographic, molecular, and clinical characteristics were cross-checked across publications, including sex, age at diagnosis, molecular subtype, major phenotypic features, author groups, and institutional affiliations. Cases considered to represent the same individual were counted only once, with data preferentially extracted from the report providing the most comprehensive clinical information and/or longest follow-up. The final pooled cohort comprised 167 patients, including 161 unique patients from the 33 eligible published studies and six newly enrolled patients from our case series. Data were independently extracted by two reviewers using a standardized form, including demographic characteristics, molecular subtype, and major clinical features such as SGA/IUGR, postnatal growth failure, hypotonia, feeding difficulties, early puberty/CPP, facial dysmorphism, speech and/or motor delay, and micropenis/cryptorchidism. Data on rhGH and/or GnRHa treatment and final adult height were extracted where available. Descriptive statistics were used to summarize the data, with categorical variables presented as frequencies and percentages. Phenotypic differences among molecular subtypes and between Asian and non-Asian populations were compared descriptively. No formal statistical testing was performed because of inconsistent variable reporting and heterogeneous data quality across the published literature.

## Results

### Clinical description

#### Case 1

A 9-year-6-month-old boy presented with testicular enlargement and pubic hair development for over six months, accompanied by voice deepening and accelerated linear growth (7 cm/year). He had a history of growth retardation during early childhood. He showed mild short stature (127.4 cm, −1.75 SDS), low weight (26 kg, −1.13 SDS), small hands and feet, Tanner stage II puberty (penile length 5 cm; testes 6 mL), and subtle dysmorphic features, including a broad prominent forehead and short fleshy-tipped nose (**Figure 1A**; **Table 1**).

**Figure 1:**
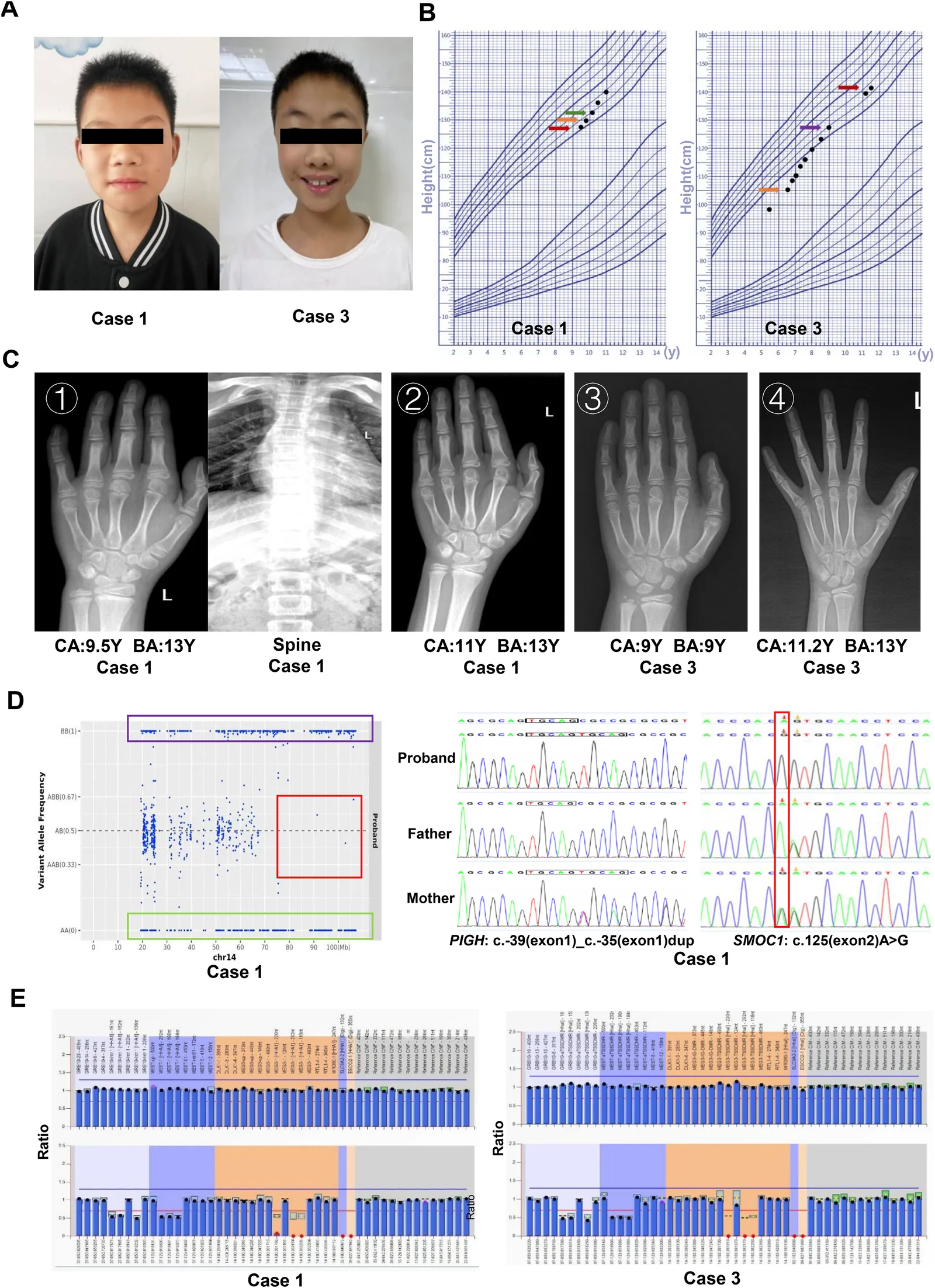
Clinical characteristics and molecular confirmation of Patients 1 and 3. **(A)** Facial features of two patients. Both exhibited a prominent nose with a fleshy nasal tip. Patient 1 demonstrated a broad and tall forehead, whereas Patient 3 showed epicanthus. **(B)** Longitudinal growth trajectories. Red arrowheads indicate initiation of GnRHa therapy; yellow arrowheads indicate initiation of rhGH therapy; purple arrowheads denote cessation of rhGH; and green arrowheads indicate the introduction of levothyroxine. Patient 1 received daily short-acting rhGH (0.15 IU/kg/day), while Patient 3 was treated with long-acting GH (0.2 mg/kg/week). Both treatments were associated with comparable annualized height velocities (8.6 cm/year [0.67 SD/year] and 8.8 cm/year [0.70 SD/year], respectively). **(C)** Bone age assessment using the Greulich–Pyle method. ① Before initiation of GnRHa therapy in Patient 1; ② After 18 months of GnRHa and rhGH treatment in Patient 1; ③ At cessation of long-acting GH therapy in Patient 3; ④ Before initiation of GnRHa therapy in Patient 3. Mild upper-thoracic scoliosis was noted in Patient 1. **(D)** WES and Sanger segregation analysis of Patient 1. WES revealed a large homozygous region on chromosome 14. The purple and green boxes highlight homozygous alternate-allele and reference-allele calls, respectively, whereas the red box highlights a region lacking heterozygous calls, suggesting loss of heterozygosity. Two homozygous variants (*PIGH* c.-39(ex1)_c.-35(ex1)dup and *SMOC1* c.125A>G) were selected for parental validation. Sanger sequencing showed paternal wild-type alleles and maternal heterozygosity, supporting maternal UPD(14). **(E)** MS-MLPA analysis of Patients 1 and 3. The assay simultaneously assessed copy number and methylation status using a healthy individual as a reference. Digestion control probes showed no post-digestion signal, confirming assay performance. All three MEG3 methylation-specific probes (MEG3-1[HHA1]-220nt, -292nt, -118nt) lacked methylation signals in both patients, indicating loss of paternal methylation. Consistent with panel D, these findings supported maternal UPD(14) in Patient 1 and indicated a 14q32 imprinting abnormality in Patient 3.

**Table 1:** Clinical manifestation and molecular characterization of the 6 patients included in this study.

Patients	Case 1	Case 2	Case 3	Case 4	Case 5	Case 6
Sex	Male	Male	Male	Male	Male	Male
Age at the genetic diagnosis	9Y6M	3M	11Y2M	3Y	3M	3Y
Gestational age (weeks)	38 ^+ 4^ w	37 w	41 ^+ 1^ w	UK	UK	UK
Birth weight SDS	−3.14	−1.98	−2.97	UK	UK	UK
Birth length SDS	UK	−1.0	−0.68	UK	UK	UK
Height SDS at first visit	−1.75	−1.45	−1.07	−2.71	−5.55	–3.7
Weight SDS at first visit	−1.13	−2.37	−0.55	−1.73	−6.22	–2.5
BMI SDS at first visit	−0.39	−1.6	+0.07	+0.09	−5.3	−0.07
Father's height (cm)	168	UK	170	UK	UK	175
Mother's height (cm)	153	UK	160	UK	UK	160
Target height (cm)	167	UK	171.5	UK	UK	174
Bone age at first visit	13Y	UK	13Y	UK	UK	UK
Pubertal stage at first visit	II	I	III	I	I	I
Reason for visit	CPP	poor feeding	short stature	speech delay	craniosynostosis	short stature
Mutation	UPD14mat	UPD14mat	paternal epimutation	paternal deletion haploinsufficiency of *DYNC1H1*	paternal deletion haploinsufficiency of *YY1*	UPD14mat homozygous *GALC* [c.1901(exon16)T>C]
Small for gestational age	+	+	+	+	+	+
Patients with CPP	+	UK	#	UK	UK	UK
Short stature/ growth retardation	+	+	+	+	+	+
Special facial features	broad forehead; short nose; fleshy nasal tip	–	epicanthus; fleshy nasal tip	epicanthus; high nasal bridge; fleshy nasal tip; large ears; micrognathia	scaphocephaly; long eyelashes; micrognathia	down-slanting eyebrows; narrow palpebral fissures
Small hands and feet	+	–	+	–	–	–
Feeding difficulties	–	+	–	–	+	+
Hypotonia	–	+	+	–	+	–
Motor delay	–	+	+	+	+	–
Speech delay	–	UK	+	+	UK	+
Other features	–	–	cryptorchidism	Autism Spectrum Disorder	craniosynostosis	–

UPD14mat, maternal uniparental disomy of chromosome 14; CPP, central precocious puberty; # rapidly progressive early puberty; SDS, standard deviation score; UK, unknown.

He was born at 38 + 4 weeks via cesarean section for oligohydramnios with low birth weight (2100 g, −3.14 SDS). Family history was unremarkable; his 12-year-old brother’s height was 146 cm (−0.81 SDS).

Routine laboratory tests were normal. Thyroid function tests revealed mildly decreased total thyroxine (TT4) at 57.53 nmol/L (reference range: 77.1–178 nmol/L) and borderline low free thyroxine (FT4) at 12.4 pmol/L (reference range: 12.5–21.5 pmol/L), with TSH remaining within the normal range. Tumor markers were negative. insulin-like growth factor-1 (IGF-1) was 307 ng/mL, appropriate for bone age (**Supplementary Table S3**). An adrenocorticotropic hormone (ACTH) stimulation test excluded adrenal insufficiency and congenital adrenal hyperplasia (**Supplementary Table S1**). A GnRH stimulation test demonstrated biochemical activation of the HPG axis with a peak LH of 23.61 IU/L, peak FSH of 10.75 IU/L, and peak LH/FSH ratio > 0.6 (**Supplementary Table S2**). Basal gonadotropins and testosterone levels are provided in **Supplementary Tables S2**, **S3**.

Imaging demonstrated advanced bone age (13 years) and mild upper-thoracic scoliosis (**Figure 1C**). Abdominal and adrenal ultrasonography were normal. Testicular ultrasound showed a longitudinal diameter of 37 mm bilaterally. Pituitary MRI revealed a pituitary height of 4.3 mm without abnormal enhancement. Together with pubertal onset before 9 years of age, biochemical HPG-axis activation, advanced bone age, accelerated growth, and bilateral testicular enlargement, these findings supported a diagnosis of CPP.

#### Case 2

A 3-month-old boy presented with poor feeding. He was born at 37 weeks by cesarean section due to placental aging and intrauterine growth restriction, with a birth weight of 2.2 kg (−1.98 SDS) and length of 47 cm (−1.0 SDS). His neonatal course included hospitalization for low birth weight, feeding difficulties, intracranial hemorrhage, and a subsequent admission for neonatal jaundice (**Table 1**). He was the second child of healthy, non-consanguineous parents.

At presentation, he exhibited weak crying, reduced auditory responsiveness, hypotonia, and poor head control. His height was 59 cm (−1.45 SDS), weight 5.16 kg (−2.37 SDS), and head circumference 38 cm (−2.14 SDS). The Gesell Developmental Schedules (GDS) demonstrated global developmental delay (gross motor 35; fine motor 28; adaptability 51; language 42; social-emotional 42).

Routine laboratory testing and metabolic screening, including acylcarnitine analysis, were normal. Brain MRI showed no significant abnormalities.

#### Case 3

An 11-year-2-month-old boy presented with short stature and RPEP. He was born at 41 + 1 weeks with a birth weight of 2.5 kg (−2.97 SDS) and length of 50 cm (−0.68 SDS), following intrauterine growth restriction characterized by absent weight gain in the final two months of gestation. According to parental recall, he showed no weight gain during the first three months of life, although no overt feeding difficulties, such as poor sucking or choking, were reported. He also exhibited hypotonia and delays in both motor and language development (**Table 1**).

He had undergone multiple evaluations for poor growth, and an externally performed GH stimulation test showed a reported peak GH of 5.29 ng/mL (**Supplementary Table S4**). Long-acting GH therapy (0.2 mg/kg/w) was initiated at 6.5 years of age for persistent short stature in the setting of SGA. Over 2.5 years, his growth velocity was approximately 10 cm, 8 cm, and 4 cm in the final half-year, with height improving from −3.1 SDS to −1.36 SDS (shown in **Figure 1B**). No adverse reactions were observed during the treatment period according to parental report. At age 3 years, he underwent laparoscopic left orchiopexy for cryptorchidism. Previous karyotype was 46,XY, and CNV analysis was normal.

At the current visit, he demonstrated accelerated growth and voice deepening. His height was 139.1 cm (−1.07 SDS) and weight 34.5 kg (−0.55 SDS). Dysmorphic features included a prominent nose with a fleshy tip and epicanthus (shown in **Figure 1A**). Pubertal assessment showed Tanner stage III with testicular volumes of 12 mL (left) and 15 mL (right). Bone age was 13 years (shown in **Figure 1C**).

Laboratory testing revealed a baseline LH of 6.64 IU/L, testosterone of 16.42 nmol/L, and IGF-1 of 211.0 ng/mL. Other biochemical tests, including complete blood count, glucose, liver and renal function, and thyroid function, were normal.

#### Case 4

A 3-year-old boy presented with speech delay. He was the third child of healthy, non-consanguineous parents, and both older sisters were healthy. He was born at term with a birth weight of 2.5 kg. At 18 months, he was diagnosed with global developmental delay (GDD), and he achieved independent walking at 2 years of age (**Table 1**).

At presentation, he remained nonverbal and exhibited growth retardation, with a height of 88.0 cm (−2.71 SDS) and weight of 12.2 kg (−1.73 SDS). Additional concerns included frequent loose stools, difficulty following instructions, poor eye contact, and minimal use of gestures (nodding, head shaking, waving), instead relying on hand-leading to communicate. These features raised suspicion for autism spectrum disorder (ASD).

Physical examination revealed subtle dysmorphic features, including epicanthus, a high nasal bridge with a fleshy nasal tip, large ears, and micrognathia.

#### Case 5

A 3-month-old boy was referred to neurosurgery for evaluation of craniosynostosis. He was the fourth child conceived via *in-vitro* fertilization to healthy, non-consanguineous parents; all three older sisters were healthy. He was born at term with a birth weight of 1.2 kg (**Table 1**).

Physical examination revealed marked dysmorphic features. He presented with scaphocephaly, characterized by an elongated anteroposterior diameter, prominent forehead, reduced biparietal width, narrowed bitemporal regions, bulging occiput, anteriorly displaced cranial vertex, and a palpable sagittal ridge. Additional abnormalities included limited extension of the right middle finger and pronounced left foot varus. Subcutaneous fat was absent, and the infant showed severe malnutrition (weight 2.5 kg, −6.22 SDS) with significant growth retardation (length 50 cm, −5.55 SDS).

#### Case 6

The last patient was a 3-year-old boy from the Children Health Care outpatient clinic of the Children’s Hospital Affiliated to Shandong University for evaluation of short stature. He was the second child of healthy, nonconsanguineous parents, born at term via cesarean section due to meconium-stained amniotic fluid. His birth weight was 1.7 kg and birth length was 42 cm, consistent with intrauterine growth restriction. During the first year of life, he experienced feeding difficulties, which contributed to failure to thrive (**Table 1**).

At the time of the visit, his height was 84 cm (–3.7  SDS) and weight was 11 kg (–2.5 SDS). The child also exhibited some delays in speech development. In addition, distinctive facial features were noted, including down-slanting eyebrows and narrow palpebral fissures.

### Genetic findings

#### Case 1

WES identified a ~ 39.58 Mb homozygous region on chromosome 14 (14q23.3–q32.33), raising suspicion of UPD. Sanger sequencing of two homozygous variants on chromosome 14 (*PIGH* c.-39(ex1)_c.-35(ex1)dup and *SMOC1* c.125A>G) showed maternal heterozygosity and paternal wild-type alleles, confirming maternal UPD (shown in **Figure 1D**). MS-MLPA further demonstrated complete loss of methylation at three *MEG3* probes that are normally paternally methylated, providing definitive evidence for maternal UPD14 (shown in **Figure 1E**).

#### Case 2

Trio-WES revealed a ~ 107.35 Mb homozygous region spanning 14p13–q32.33. Pedigree analysis suggested a maternal origin, which was confirmed by MS-MLPA (shown in **Supplementary Figures 1A, B**).

#### Case 3

WES detected no pathogenic CNVs, UPD, structural variants, or SNVs. However, MS-MLPA demonstrated loss of paternal methylation in the 14q32.2 imprinted region, consistent with TS14 due to a paternal epimutation (shown in **Figure 1E**).

#### Case 4

WES identified a heterozygous ~ 2.0 Mb deletion at 14q32 (chr14:101193405–103192843), encompassing *TECPR2*, *DYNC1H1*, *DLK1*, and *RTL1*(shown in **Supplementary Figure 1C**). qPCR confirmed a 50% reduction in *DYNC1H1* dosage, whereas both parents showed normal dosage, indicating a *de novo* heterozygous deletion. This variant is associated with *DYNC1H1*-related neurodevelopmental disorder, characterized by variable degrees of motor, speech, and/or cognitive delay, which may partially explain the patient’s phenotype. MS-MLPA confirmed a paternal deletion (shown in **Supplementary Figure 1D**).

#### Case 5

Trio-WES detected a heterozygous ~ 1.71 Mb deletion at 14q32.2 (chr14:99640487–101351125) (shown in **Supplementary Figure 1E**). MS-MLPA confirmed a paternal deletion (shown in **Supplementary Figure 1F**). The deleted region included *YY1*, associated with Gabriele-de Vries syndrome, an autosomal dominant neurodevelopmental disorder involving developmental delay, cognitive impairment, feeding difficulties, and dysmorphic features. Both parents were wild-type, indicating a *de novo* variant, which likely contributed to the patient’s more severe phenotype.

#### Case 6

WES identified a ~ 76.24 Mb homozygous region on 14q11.2–q32.33. Sanger sequencing demonstrated maternal heterozygosity and paternal wild-type alleles for *GALC* c.1901T>C, while the patient was homozygous, supporting maternal UPD (shown in **Supplementary Figure 1G**). *GALC* mutations cause Krabbe disease, and c.1901T>C is typically associated with the late-onset form, characterized by gait disturbance, cognitive decline, peripheral neuropathy, and visual impairment. MS-MLPA further confirmed maternal UPD14 (shown in **Supplementary Figure 1H**).

### Follow-up

Patient 1 received GnRHa therapy for CPP, followed by rhGH at 0.15 IU/kg/day (0.05 mg/kg/day) for height optimization in the context of CPP with markedly advanced bone age and TS14-associated growth vulnerability; no GH stimulation test was performed before GH initiation. During follow-up, FT4 initially normalized after 1 month of rhGH therapy without thyroid hormone replacement. Mild hypothyroxinaemia subsequently recurred after 4 months of rhGH treatment (FT4 11.7 pmol/L), while TSH remained within the normal range. This was considered in the context of GH-related thyroid axis changes or possible mild central hypothyroidism and was corrected with levothyroxine (12.5 μg/day) (**Supplementary Table S3**). After 18 months of GnRHa and 14 months of rhGH treatment, height increased from 127.4 cm (–1.75 SDS) to 140.3 cm (–0.75 SDS), with an annualized growth velocity of 8.6 cm/year (shown in **Figure 1B**). Apart from recurrent mild hypothyroxinaemia requiring low-dose levothyroxine treatment, pubertal progression and bone age advancement remained well controlled. No injection-site reactions, symptoms suggestive of intracranial hypertension, clinically apparent scoliosis progression, or other treatment-related adverse events were observed during follow-up (shown in **Figure 1C**; **Supplementary Table S3**).

Patient 2, at six months of age, demonstrated improved feeding and developmental scores (GDS 75–90–89–78–83), with a height of 65.6 cm (–1.29 SDS), weight of 6.75 kg (–2.07 SDS), and head circumference of 41.3 cm (–1.72 SDS).

Patient 3 was treated with GnRHa for RPEP; therapy is ongoing with stable pubertal suppression.

At the last follow-up (5 years 5 months), Patient 4 remained nonverbal, had difficulty understanding complex instructions, and struggled with peer interactions. He communicated only through simple gestures, while motor ability and muscle strength were reported as normal by his parents.

Unfortunately, Patient 5 died after the second surgery due to cardiac and respiratory arrest secondary to milk aspiration. Although no direct relationship with TS14-specific treatment was identified, this fatal event should be viewed in light of his severe clinical phenotype, profound growth retardation, severe malnutrition, and possible feeding difficulties, which may have contributed to an increased risk of aspiration.

At the last follow-up (5 years 11 months), Patient 6 continued to exhibit short stature, with a height of 102 cm (−3.3 SDS), but showed markedly improved appetite and gained 10 kg over the preceding year, reaching 20 kg (−0.53 SDS). Motor and speech development were reported as normal by his parents. Growth hormone therapy had not been initiated by the last follow-up because the family did not wish to proceed with treatment at that time.

### Literature review

A total of 167 patients with TS14, including six from the present study, were identified from published cohorts and case reports (1, 3, 5–35). The male-to-female ratio was approximately equal (77: 90), and the age at molecular diagnosis ranged from 1 month to 33 years, reflecting broad phenotypic variability. The most frequent clinical features were CPP, hypotonia, SGA, and distinctive facial appearance, with only minor variation between the UPD14mat and epimutation subgroups. Speech and motor delays, together with facial dysmorphism, were more prominent among patients with deletions (shown in **Figure 2**; **Supplementary Table S5**).

**Figure 2:**
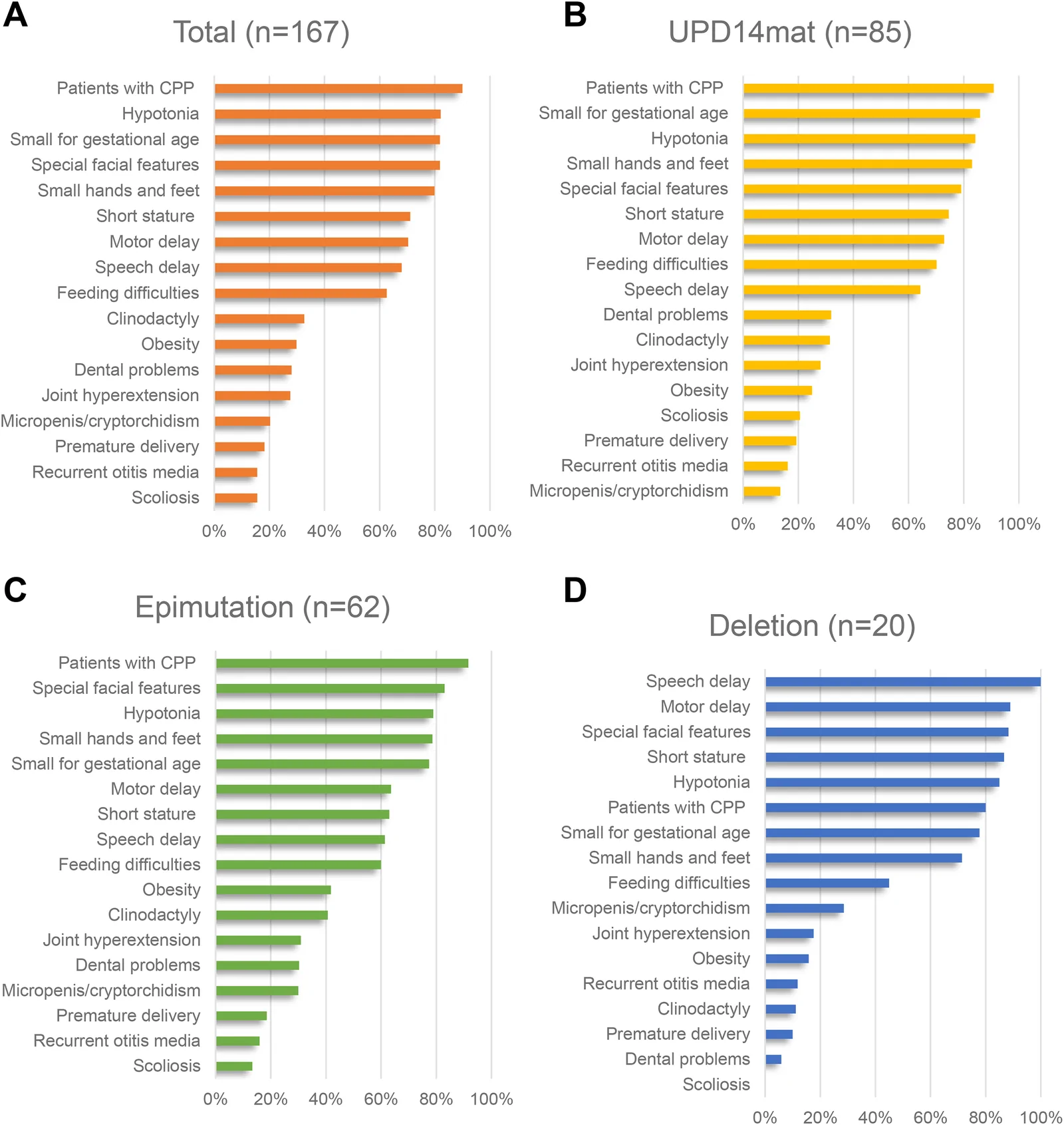
Clinical features reported in 167 cases of Temple syndrome, subdivided by (epi)genotype. Not all clinical features were reported for every patient; therefore, percentages were calculated based on cases in which the presence or absence of each feature could be reliably determined. UPD14mat, maternal uniparental disomy of chromosome 14.

Descriptive comparisons between Asian and non-Asian cohorts showed no major differences in the principal clinical manifestations or genotypes. However, Asian patients were more frequently male and showed lower reported frequencies of facial dysmorphism and obesity but a higher reported frequency of micropenis and/or cryptorchidism (**Table 2**).

**Table 2:** Clinical features in 167 cases of Temple syndrome compared between Asian and non-Asian populations.

Patients	Asia (n= 69)	Non-Asia (n= 98)
Genotype (UPD14mat: Epimutation: Deletion)	38:24:7	47:38:13
Sex ratio (male: female)	41:28	36:62
Intrauterine growth retardation	100%	100%
Premature delivery (<37weeks)	11/64 (17.19%)	9/46 (19.57%)
Small for gestational age	62/69 (89.86%)	73/96 (76.04%)
Patients with CPP	38/45 (84.44%)	43/45 (95.56%)
Short stature	44/50 (88.00%)	47/78 (60.26%)
Special facial features	40/62 (64.52%)	77/81 (95.06%)
Small hands and feet	43/59 (72.88%)	60/70 (85.71%)
Feeding difficulties	40/64 (62.50%)	52/83 (62.65%)
Joint hyperextension	14/61 (22.95%)	18/55 (32.73%)
Scoliosis	13/65 (20.00%)	8/70 (11.43%)
Hypotonia	46/60 (76.67%)	60/69 (86.96%)
Recurrent otitis media	13/65 (20.00%)	8/70 (11.43%)
Irregular or crowded teeth/dental problems	26/62 (41.94%)	11/70 (15.71%)
Fifth finger clinodactyly/ Clinodactyly	22/65 (33.85%)	26/82 (31.71%)
Micropenis and/or cryptorchidism	11/40 (27.50%)	2/24 (8.33%)
Obesity	12/69 (17.39%)	34/85 (40.00%)
Motor delay	35/55 (63.64%)	36/46 (78.26%)
Speech delay	30/50 (60.00%)	38/50 (76.00%)

Not all clinical features were documented for every case. Accordingly, the proportions shown in this table were calculated only from cases in which clinical features could be unequivocally classified as present or absent. UPD14mat, maternal uniparental disomy of chromosome 14; CPP, central precocious puberty.

Three of the six patients in our series harbored additional molecular findings, prompting us to review five previously reported TS14 cases with additional molecular findings or diagnoses (**Table 3**) (5–7, 36, 37). The molecular backgrounds of these cases were heterogeneous, including additional variants or dosage effects associated with regions affected by UPD or deletion, an additional molecular diagnosis unrelated to TS14, multilocus imprinting disturbance (MLID), and a *ZNF445*-related imprinting defect. As summarized in **Table 3**, the clinical features were classified as consistent with classical TS14, potentially attributable to the additional molecular finding, or as having overlapping or uncertain attribution.

**Table 3:** Temple syndrome with additional molecular findings: clinical features and probable phenotypic attribution.

Patients	Case 4	Case 5	Case 6	Kagami et al.2021, Japan#	Grosvenor et al., 2022, UK	Chaisrisawadisuk et al., 2023, Thailand*	Zubair et al., 2025, USA	Sá et al., 2025, Portugal
Sex	Male	Male	Male	Female	Male	Female	Male	Male
Molecular mechanism of TS14	paternal deletion approximately 2.0Mb	paternal deletion approximately 1.71Mb	UPD14mat	epimutation	epimutation	14q32 deletion approximately 9.05 Mb	UPD14mat	epimutation
Features consistent with classical TS14	SGA Postnatal growth retardation Fleshy nasal tip Micrognathia ASD-like features† GDD†	SGA Postnatal growth retardation Feeding difficulties Hypotonia Motor delay Micrognathia	IUGR, SGA Postnatal growth retardation Feeding difficulties Speech delay	SGA Postnatal growth retardation Hypotonia Feeding difficulties Small hands Prominent forehead Relative macrocephaly GDD	Prominent forehead Relative macrocephaly Broad nasal tip Flat nasal bridge Speech delay	SGA Hypotonia Feeding difficulties Small hands and feet Broad nasal root Micrognathia High arched palate	IUGR, SGA Postnatal growth retardation Frontal bossing Micrognathia Triangular face GDD† Hypotonia† Feeding difficulties	IUGR† Triangular face Frontal bossing Prominent forehead Macrocephaly Depressed nasal root Wide nasal bridge High arched palate Hypotonia Feeding difficulties
Additional molecular finding	haploinsufficiency of *DYNC1H1*	haploinsufficiency of *YY1*	homozygous *GALC* [c.1901T>C, p.Leu634Ser]	homozygous *ZNF445* [c.2803C>T, p.Gln935*]	hypomethylation of *KCNQ1OT1* TSS DMR	candidate genes *YY1 / BCL11B*	homozygous *TECPR2* [c.1319del, p.Leu440Argfs*19]	homozygous *RFX6* [c.541C>T, p.Arg181Trp]
Features potentially attributable to the additional molecular finding	ASD-like features† GDD†	Craniosynostosis Scaphocephaly	No clinically apparent *GALC*-related manifestations at the latest evaluation	Typical SRS features and PWS-like marked hypotonia	Lateralized overgrowth/ body asymmetry Facial nevus simplex	Left anterior plagiocephaly Scaphocephaly Craniosynostosis	GDD† Hypotonia† Areflexia of lower limbs Behavioral dysregulation Ataxic gait Impaired pain sensitivity Dysphagia Recurrent infections Strabismus	IUGR† Neonatal diabetes Intestinal atresia Gallbladder hypoplasia Meckel diverticulum
Features of overlapping or uncertain attribution	Epicanthus Large ears High nasal bridge	Long eyelashes	Down-slanting eyebrows Narrow palpebral fissures	–	–	Pulmonary stenosis Right ureterocele Abnormal ears	Epicanthus	Posteriorly rotated low-set ears Long horizontal palpebral fissures

TS14, Temple syndrome; UPD14mat, maternal uniparental disomy of chromosome 14; IUGR, intrauterine growth retardation; SGA, small for gestational age; ASD, autism spectrum disorder; GDD, global development delay; SRS, Silver-Russell syndrome; PWS, Prader-Willi syndrome.

# The homozygous *ZNF445* variants could be responsible for the severely hypomethylated *MEG3/ DLK1*:IG-DMR (CG-A) and *MEG3*:TSS-DMR and the mild-to-moderate degrees of multilocus imprinting disturbance (MLID) of the patient. * The parental origin of the patient’s deleted chromosome was not determined; it was likely that the patient had a paternal deletion of chromosome 14q32 according to her clinical manifestations. The chromosome 14q32 deletion region encompassed *BCL11B* and *YY1* genes, which were candidate genes causing craniosynostosis. †Features potentially shared between TS14 and the additional molecular diagnosis; definitive exclusive phenotypic attribution cannot be made.

Growth failure—both prenatal and postnatal—remains a defining feature of TS14. Among 14 patients (excluding Japanese data) with recorded final height, the median adult height was –3.15 SDS. The epimutation subgroup showed the greatest impairment (median –3.6 SDS). In our exploratory descriptive comparison, patients who received rhGH and/or GnRHa had a higher median final height (median –1.88 SDS) than untreated individuals (median –3.3 SDS) (**Table 4**; **Supplementary Table S6**).

**Table 4:** Final height SDS of patients from the literature after excluding Japanese data.

Patients	Total	Genotype			Therapy	
		UPD14mat	Epimutation	Deletion	Treatment	Untreatment
Number	14	5	5	4	7	7
Final height SDS (median and range)	−3.15 (−4.76 ~ −0.76)	−1.88 (−4 ~ −0.76)	−3.6 (−4.76 ~ −0.8)	−2.53 (−4.26 ~ −0.99)	−1.88 (−4.76 ~ −0.76)	−3.3 (−4 ~−0.99)

Treatment, with rhGH and/or GnRHa treatment; Untreatment, without rhGH and/or GnRHa treatment.

## Discussion

Temple syndrome (TS14) is a markedly under-recognized imprinting disorder owing to its broad phenotypic variability and substantial overlap with other imprinting conditions. Before this study, only four pediatric cases had been reported from China, highlighting a substantial diagnostic gap and limited regional awareness (8–11). By presenting the largest Chinese pediatric series to date and integrating findings from 167 published cases, our study enhances the clinical characterization of TS14 and highlights diagnostic clues that may facilitate earlier recognition.

In our review of 167 patients, including six from our series, the major clinical manifestations—SGA, postnatal growth failure, hypotonia, early-onset puberty, feeding difficulties, developmental delay, and characteristic facial features—were broadly consistent across populations. Although the core clinical manifestations did not differ substantially between populations, Asian patients were more frequently male and showed lower reported frequencies of facial dysmorphism and obesity but a higher reported frequency of micropenis and/or cryptorchidism (15). These differences may reflect variations in diagnostic practices, clinical evaluation protocols, and reporting patterns.

Our findings reaffirm that subtle characteristic facial features—particularly a broad forehead and a fleshy nasal tip—serve as valuable diagnostic clues. These features have been frequently described in Western cohorts (1) but rarely emphasized in Japanese reports (15, 23, 25–28, 30). In our series, they were observed in three of the six patients and have also been documented in two previously reported Chinese cases (8, 10). Notably, these facial traits appear to persist beyond early childhood, even as other SRS-like characteristics tend to become less pronounced with age.

A notable finding of our study was the presence of additional molecular abnormalities in three of the six patients. These cases were considered separately in delineating the classical TS14 phenotype because the additional findings in Cases 4 and 5 may have contributed to their atypical features, raising the possibility of blended phenotypes. In Case 4, the likely pathogenic *DYNC1H1* deletion may have contributed to the ASD-like features and GDD, although definitive attribution remains difficult because of phenotypic overlap with TS14 (38, 39). In Case 5, we identified a paternal deletion encompassing *YY1* in a patient with craniosynostosis. This finding is consistent with a previously reported case in which the parental origin of the 14q32 deletion was undetermined and supports a possible contribution of *YY1* haploinsufficiency to craniosynostosis (37). In contrast, at the latest evaluation, there was no clinical evidence that the *GALC* finding in Case 6 contributed to the current phenotype (40). Collectively, these observations suggest that atypical manifestations in some patients may reflect the contribution of an additional molecular finding or diagnosis and should be interpreted cautiously before being considered part of the expanded phenotypic spectrum of TS14.

Definitive diagnosis of TS14 requires molecular confirmation. Consistent with current recommendations, MS-MLPA is the preferred first-line test for suspected TS14 because it enables simultaneous assessment of methylation status and copy-number changes at the 14q32 imprinted region (15). In our series, WES was initially performed in patients whose atypical presentations did not immediately suggest an imprinting disorder. Trio-based WES, performed in two cases, also facilitated assessment of the parental origin of UPD and deletions. Overall, WES identified regions of homozygosity suggestive of UPD in three of the six patients, large 14q32 deletions in two, and additional pathogenic variants in three, demonstrating its value in detecting TS14-related abnormalities and co-occurring genetic findings that may modify the phenotype. However, WES findings alone were considered suggestive rather than diagnostic. In Case 3, WES was negative, whereas subsequent MS-MLPA confirmed an abnormal methylation pattern at the 14q32 imprinted region. This case illustrates that a negative WES result does not exclude TS14 and that methylation-specific testing should be pursued when clinical suspicion persists. Accordingly, TS14 should be considered in children presenting with SGA/IUGR, postnatal growth failure, hypotonia, feeding difficulties, and early-onset puberty. Methylation-specific testing of the 14q32 imprinted region is required for definitive confirmation, whereas WES may serve as a broader etiological screening tool in children with unexplained SGA or atypical presentations.

Growth impairment is a hallmark of TS14, and final adult height is frequently compromised. This burden is further amplified by the high prevalence of CPP, which accelerates bone maturation and restricts prepubertal growth. While earlier reports estimated a median final height of −2.04 SDS (1), untreated individuals with available final-height data in our review had a median final height of −3.3 SDS. However, this estimate was based on a small, selected subset and should be interpreted cautiously. Short-term rhGH treatment has been reported to improve growth outcomes, particularly when initiated before puberty (12–14). Brightman et al. reported that six patients who initiated rhGH at a median age of 3.3 years (range: 2.3–4.1years) achieved a median height improvement of 1.31 SDS and an increase in height velocity of 5.30 cm/year after 1-year treatment (14).

In patients with CPP, combined rhGH and GnRHa therapy may help preserve height potential. However, responses vary considerably and appear influenced by baseline stature, pubertal progression, and underlying genetic mechanisms (13, 15). Long-term follow-up by Juriaans et al. demonstrated a mean height gain of 1.76 SDS and a median adult height of −0.67 SDS among patients receiving prolonged rhGH therapy, most of whom also received GnRHa (13). A recent Japanese cohort similarly showed an improvement from −3.4 to −2.4 SDS at near-final height following rhGH, with or without GnRHa (15). Apparent inconsistencies, such as untreated individuals achieving a near-final height of −2.1 SDS in the same study, may reflect selection bias because patients with more severe baseline short stature were more likely to receive treatment. In our exploratory analysis, treated patients had a higher median final height than untreated patients (−1.88 SDS vs. −3.3 SDS), and target height was associated with outcome (**Supplementary Table S6**). Nevertheless, this unadjusted comparison was based on a small, selected sample and may have been confounded by treatment indication, baseline height, age at diagnosis, pubertal timing, and other potential confounders.

Our case series provides additional real-world observations on the growth outcomes of hormonal treatment in TS14. Patient 1 showed a clinically meaningful increase in height SDS (ΔSDS +1.0) during combined treatment with GnRHa and rhGH. This response cannot be attributed specifically to rhGH and may reflect the combined effects of GnRHa-mediated pubertal suppression, rhGH therapy, thyroid hormone replacement, altered bone age progression, and natural growth variation. Moreover, treatment was initiated after substantial skeletal maturation and pubertal activation had already occurred, with a bone age of 13 years at a chronological age of 9.5 years, limiting interpretation of the potential benefit for final adult height. Patient 3 showed growth acceleration during long-acting GH therapy. Nevertheless, given the small sample size, observational design, combined treatment in patient 1, and limited follow-up, these data do not establish the efficacy or safety of rhGH, long-acting GH, or GnRHa in TS14. Rather, they provide preliminary clinical observations that may inform future prospective studies of treatment timing, long-term growth outcomes, and safety.

In conclusion, our study broadens the clinical characterization of TS14 in Chinese children and suggests that additional molecular findings may contribute to atypical or blended presentations, underscoring the value of comprehensive genomic testing. Early recognition may facilitate endocrine monitoring and individualized treatment, but the optimal timing of GH/GnRHa therapy, long-term safety, and ultimate height benefit remain to be determined in future prospective studies.

## Data Availability

The clinical and molecular findings supporting the conclusions of this study are presented in the article and its Supplementary Material. The genetic findings were obtained from clinical genetic testing reports issued by hospitals or third-party diagnostic laboratories. The raw WES data were not provided to the authors. The complete individual genetic testing reports and underlying clinical records are not publicly available due to patient privacy and confidentiality considerations. Further inquiries may be directed to the corresponding author, subject to applicable ethical and institutional restrictions.

## Supplementary material

Supplementary Figure 1 Gene validation of Patient 2, Patient 4-6. **(A,B)** Gene validation of Patient 2. **(A)** Trio-WES detected a large homozygous region on chromosome 14 in the proband, and pedigree analysis indicated maternal UPD. **(B)** The specifically designed MS-MLPA assay showed no methylation signals were detected on the three probes in the imprinted gene region which should be methylated on the paternal allele, providing strong molecular evidence for maternal UPD. **(C,D)** Gene validation of Patient 4. **(C)** WES detected a heterozygous deletion region on chromosome 14q32 in the proband. **(D)** MS-MLPA assay showed a heterozygous deletion involving the 14q32 and no methylation signals on the paternal allele, confirming the paternal deletion. **(E,F)** Gene validation of Patient 5. **(E)** Trio-WES detected a heterozygous deletion region on chromosome 14q32 in the proband. **(F)** MS-MLPA assay showed the paternal deletion on chromosome 14q32. **(G,H)** Gene validation of Patient 6. **(G)** WES detected a large homozygous region on chromosome 14 in the proband, Sanger sequencing demonstrated that the patient’s mother was heterozygous for *GALC* [c.1901(exon16)T>C], whereas the father carried wild-type alleles, and the patient was homozygous for the variant, considering maternal UPD. **(H)** MS-MLPA assay indicated the maternal UPD on chromosome 14.
