# Supplementary material for: Temple syndrome in Chinese children: a case series and literature review
Source: Front Endocrinol (Lausanne). 2026 Oct 19;17:1871637. doi: 10.3389/fendo.2026.1871637 (PMC13724377; doi:10.3389/fendo.2026.1871637)
Supplement: Supplementary file 1 [file SupplementaryFile1.pdf]

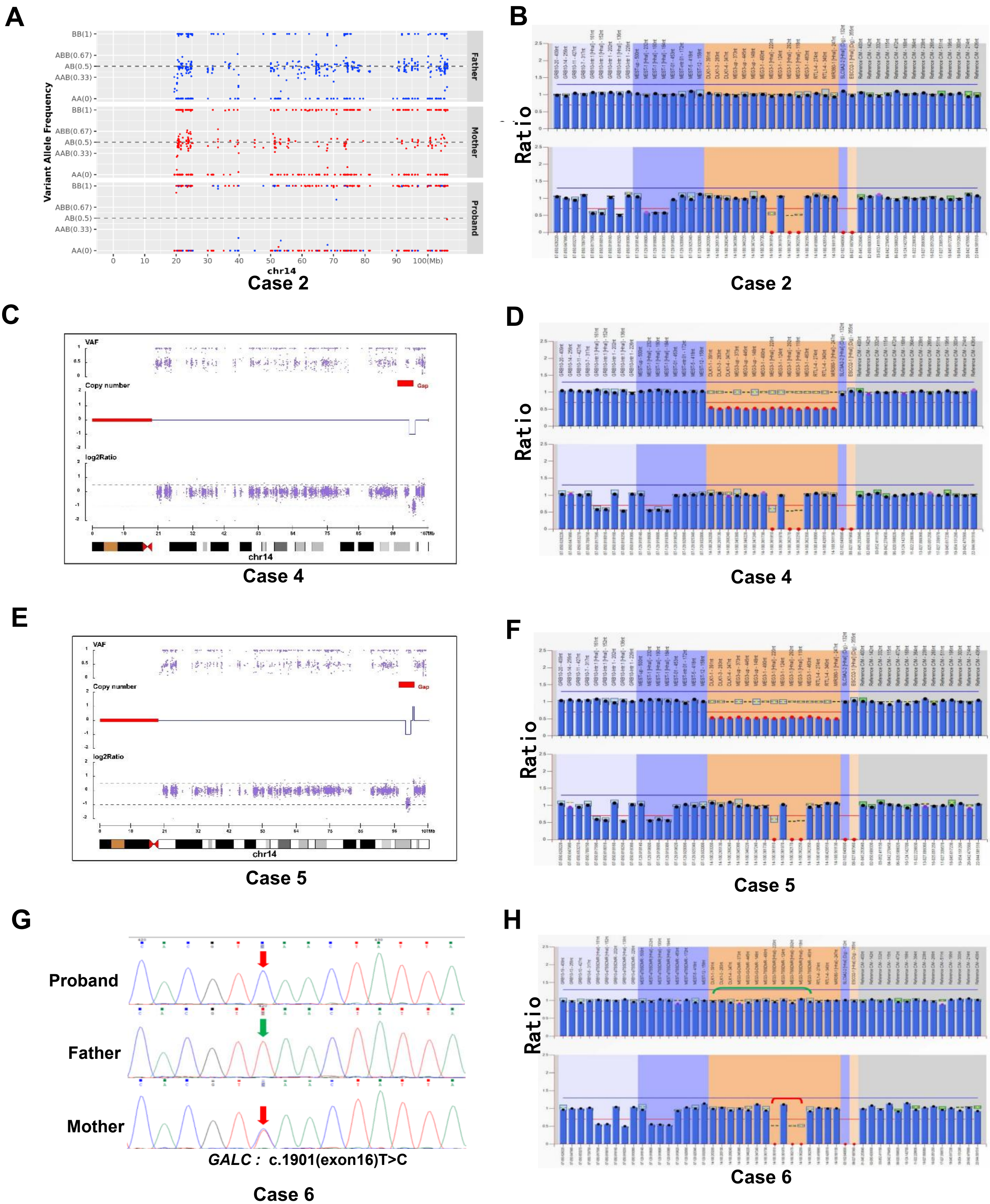

A
B
C
D
E
F
G
H
Variant Allele Frequency
BB(1)
ABB(0.67)
AB(0.5)
AAB(0.33)
AA(0)
Father
Mother
Proband
chr14
0 10 20 30 40 50 60 70 80 90 100(Mb)
Case 2
Ratio
Case 2
VAF
Copy number
Gap
log2Ratio
chr14
Case 4
Ratio
Case 4
VAF
Copy number
Gap
log2Ratio
chr14
Case 5
Ratio
Case 5
Proband
Father
Mother
GALC : c.1901(exon16)T>C
Case 6
Ratio
Case 6
