# Supplementary material for: Temple syndrome in Chinese children: a case series and literature review
Source: Front Endocrinol (Lausanne). 2026 Oct 19;17:1871637. doi: 10.3389/fendo.2026.1871637 (PMC13724377; doi:10.3389/fendo.2026.1871637)
Supplement: Supplementary file 2 [file Table1.docx]

**Table S1.** ACTH stimulation test results of the patient 1.

| Hormones | 0'min | 30'min | 60'min |
| --- | --- | --- | --- |
| Cortisol (nmol/L) | 220.60 | 698.90 | 810.60 |
| 17-OHP (ng/mL) | 0.38 | 1.90 | 2.00 |
| DHEA-S (ug/dL) | 93.84 | 89.87 | 96.79 |
| ASD (ng/mL) | 0.73 | 1.07 | 0.99 |

ACTH, adrenocorticotropic hormone; 17-OHP, 17-hydroxyprogesterone; DHEA-S, dehydroepiandrosterone sulfate; ASD, androstenedione.

**Table S2.** GnRH stimulation test results of the patient 1.

| Hormones | 0'min | 30'min | 60'min | 90'min |
| --- | --- | --- | --- | --- |
| FSH (lU/L) | 6.14 | 9.2 | 10.75 | 9.81 |
| LH (lU/L) | 4.03 | 23.61 | 23.29 | 18.06 |

GnRH, gonadotropin-releasing hormone; FSH, follicle-stimulating hormone; LH, luteinizing hormone.

**Table S3.** Hormonal characteristics before and after treatment of the patient 1.

| Hormones | basal | 3 months | 5 months  GH-1 month | 8 months  GH-4 months | 11 months  GH-7 months | 18 months  GH-14 months |
| --- | --- | --- | --- | --- | --- | --- |
| Peak LH (IU/L) | 23.61 | 1.75 | / | / | / | / |
| Peak FSH (IU/L) | 10.75 | 1.37 | / | / | / | / |
| T (nmol/L) | 11.85 | <0.087 | / | / | / | / |
| E2 (pmol/L) | 52.05 | <18.35 | / | / | / | / |
| P (nmol/L) | 0.679 | 0.462 | / | / | / | / |
| HCG (IU/L) | <0.10 | / | / | / | / | / |
| AFP (ng/mL) | 0.897 | / | / | / | / | / |
| FT3 (pmol/L) | 5.61 | / | 6.77 | 6.45 | 6.00 | 5.70 |
| FT4 (pmol/L) | 12.40 | / | 13.70 | 11.70 | 13.30 | 14.40 |
| TSH (IU/L) | 1.35 | / | 1.72 | 1.59 | 1.37 | 0.9 |
| FBG (mmol/L) | 4.22 | / | 4.93 | 4.65 | 5.16 | 5.09 |
| IGF-1 (ng/mL) | 307.0 | / | 541.0 | 428.0 | 540.7 | 475.0 |

LH, luteinizing hormone; FSH, follicle-stimulating hormone; T, testosterone; E2, estradiol; P, progesterone; HCG, human chorionic gonadotropin; AFP, alpha-fetoprotein; FT3, free tri-iodothyronine; FT4, free thyroxine; TSH, thyroid-stimulating hormone; FBG, fasting blood glucose; IGF-1, insulin-like growth factor 1.

**Table S4.** GH stimulation test results of the patient 3 at an external hospital.

|  | 0'min | 30'min | 60'min | 90'min | 120'min |
| --- | --- | --- | --- | --- | --- |
| Arg (ng/mL) | 0.16 | 1.23 | 5.29 | 1.29 | 0.31 |
| Clo (ng/mL) | 1.11 | 1.41 | 3.06 | 2.72 | 1.54 |

Arg, GH levels after arginine stimulation; Clo, GH levels after clonidine stimulation; GH, growth hormone.

Table S5. Clinical features frequently reported in 167 cases of Temple syndrome, subdivided by (epi)genotype

| Patients | Total (n=167) | UPD14mat (n=85) | Epimutation (n=62) | Deletion (n=20) |
| --- | --- | --- | --- | --- |
| Premature delivery | 20/110 (18.18%) | 11/57 (19.30%) | 8/43 (18.60%) | 1/10 (10%) |
| Small for gestational age | 135/165 (81.82%) | 73/85 (85.88%) | 48/62 (77.42%) | 14/18 (77.77%) |
| Patients with CPP | 81/90 (90.00%) | 40/44 (90.91%) | 33/36 (91.67%) | 8/10 (80%) |
| Short stature | 91/128 (71.09%) | 44/59 (74.58%) | 34/54 (62.96%) | 13/15 (86.67%) |
| Special facial features | 117/143 (81.82%) | 53/67 (79.10%) | 49/59 (83.05%) | 15/17 (88.24%) |
| Small hands and feet | 103/129 (79.84%) | 49/59 (83.05%) | 44/56 (78.57%) | 10/14 (71.43%) |
| Feeding difficulties | 92/147 (62.59%) | 47/67 (70.15%) | 36/60 (60.00%) | 9/20 (45%) |
| Joint hyperextension | 32/116 (27.59%) | 16/57 (28.07%) | 13/42 (30.95%) | 3/17 (17.65%) |
| Scoliosis | 21/135 (15.56%) | 15/73 (20.55%) | 6/45 (13.33%) | 0/17 (0%) |
| Hypotonia | 106/129 (82.17%) | 48/57 (84.21%) | 41/52 (78.85%) | 17/20 (85%) |
| Recurrent otitis media | 21/135 (15.56%) | 12/74 (16.22%) | 7/44 (15.91%) | 2/17 (11.76%) |
| Irregular or crowded teeth/dental problems | 37/132 (28.03%) | 23/72 (31.94%) | 13/43 (30.23%) | 1/17 (5.88%) |
| Fifth finger clinodactyly/ Clinodactyly | 48/147 (32.65%) | 22/70 (31.43%) | 24/59 (40.68%) | 2/18 (11.11%) |
| micropenis and/or cryptorchidism | 13/64 (20.31%) | 5/37 (13.51%) | 6/20 (30.00%) | 2/7 (28.57%) |
| Obesity | 46/154 (29.87%) | 20/80 (25%) | 23/55 (41.82%) | 3/19 (15.79%) |
| Motor delay | 71/101 (70.30%) | 35/48 (72.92%) | 28/44 (63.64%) | 8/9 (88.89%) |
| Speech delay | 68/100 (68.00%) | 27/42 (64.29%) | 27/44 (61.36%) | 14/14 (100%) |

**Tabl**e S6. Final height SDS of patients in literature.

| Patients | Sex | Nationality | Precocious puberty | GnRHa | rhGH | Final height (SDS) | Target height  (cm) | Genotypes | Refenence  (PMID) |
| --- | --- | --- | --- | --- | --- | --- | --- | --- | --- |
| P1 | Female | Netherlands | + | + | + | –0.76 | / | UPD14mat | 36362517 |
| P2 | Female | Netherlands | + | + | + | –1.50 | / | UPD14mat | 36362517 |
| P3 | Female | Hong Kong | + | + | + | –1.88 | / | UPD14mat | 27383220 |
| P4 | Female | Bulgaria | + | + | - | –4.76 | 151.0 | Epimutation | 36728278 |
| P5 | Female | France | + | + | - | –3.3 | 159.0 | UPD14mat | 29659920 |
| P6 | Female | France | + | - | - | –4.0 | 157.7 | UPD14mat | 29659920 |
| P7 | Female | France | + | - | - | –3.3 | 161.5 | Epimutation | 29659920 |
| P8 | Female | France | - | - | - | –3.6 | 157.5 | Epimutation | 29659920 |
| P9 | Male | France | + | + | - | –0.8 | 191.0 | Epimutation | 29659920 |
| P10 | Male | France | - | - | - | –3.9 | 174.5 | Epimutation | 29659920 |
| P11 | Female | Italy | + | - | - | –0.99 | 169.5 | Deletion | 26333654 |
| P12 | Female | Italy | + | - | - | –2.99 | 159.5 | Deletion | 26333654 |
| P13 | Female | Italy | + | - | - | –2.07 | 159.0 | Deletion | 26333654 |
| P14 | Male | Italy | + | + | - | –4.26 | / | Deletion | 26333654 |
